# Association between mobile technology use and child adjustment in early elementary school age

**DOI:** 10.1371/journal.pone.0199959

**Published:** 2018-07-25

**Authors:** Rikuya Hosokawa, Toshiki Katsura

**Affiliations:** 1 School of Nursing, Nagoya City University, Nagoya, Japan; 2 Graduate School of Medicine, Kyoto University, Kyoto, Japan; Karolinska Institutet, SWEDEN

## Abstract

The time that children spend using digital devices is increasing rapidly with the development of new portable and instantly accessible technology, such as smartphones and digital tablets. Although prior studies have examined the effects of traditional media on children’s development, there is limited evidence on the impact of mobile device use. The current study aimed to clarify the link between mobile device use and child adjustment. The sample included 1,642 children aged 6 in first grade at elementary schools in Japan. Parents completed a self-report questionnaire regarding their children’s use of mobile devices and emotional/behavioral adjustment. We performed inverse probability of treatment weighted (IPTW) logistic regression to compute odds ratios (OR) for emotional/behavioral problems according to mobile device use. The values for IPTW analysis were computed based on variables assessing sociodemographics and child characteristics. Among the participants, 230 (14.0%) were regular users (60 minutes or more on a typical day) and 1,412 (86.0%) non-regular users (under 60 minutes on a typical day). Relative to non-regular use, regular use of mobile devices was significantly linked to conduct problems (IPTW-OR: 1.77, 95% CI: [1.03–3.04], *p* < .05) and hyperactivity/inattention (IPTW-OR: 1.82, 95% CI: [1.15–2.87], *p* < .01). Based on these results, routine and frequent use of mobile devices appear to be associated with behavioral problems in childhood.

## Introduction

The time that children spend using digital devices is increasing rapidly with the development of new portable and instantly accessible technology, such as smartphones and digital tablets. Furthermore, with the dramatically rapid development of media games, learning packages, and educational applications for young children, opportunities for using mobile devices have been growing, children’s usage time has become increasingly longer, and child target users of mobile devices are becoming younger [1,2,3,4]. In Japan, the amount of time that children spend using mobile devices has also increased dramatically. A recent survey found that, according to the Japan Ministry of Education, the proportion of children using mobile devices for over an average of 1 hour per day was 15% among elementary schoolers and 48% among junior high schoolers [5]. Children can use mobile devices anytime and anywhere for various purposes, such as playing games, doing schoolwork, chatting with friends, and surfing the internet. From traditional media like television and video games to new media including not only home computers but also mobile devices, such as smartphones and digital tablets, media are an increasingly dominant force in children’s lives [4,6]. Media devices are expected to play an increasing role in daily life, even among young children. The increasing amount of time that children spend using mobile devices has raised concerns about the influence of digital technology use on the health of developing children.

Several studies have suggested that the impact of computer use on children’s development can be positive or negative, depending on the context of use. While computer use can be positively related to cognitive and academic skills [7–11], it can be negatively related to social and psychological development. For example, frequent computer use increases children’s social isolation, robs children of time for social activities with others, and interferes with social development [12,13]. In addition, frequent computer use may increase children’s social isolation resulting in depression and loneliness [14,15]. Furthermore, time spent using media (including both traditional and new media), can displace time used for quality parent-child interaction, such as sharing enriching experiences and activities Thus, increased media exposure is likely to be associated with reduced parent–child interaction, including shared reading and playing together with toys, which reduces opportunities for verbal interaction with parents [16,17,18]. Many studies have suggested that the reduced parent–child verbal interactions is associated with negative developmental outcomes, including language development, self-regulation and later academic achievement [19,20,21,22]. Similarly, time spent using media can reduce the time children spend playing with peers. Playing is an important element of childhood, which supports the development of problem-solving skills and creative expression [23]. As frequent media use is likely to reduce children’s playtime with peers and engaging in creative play, it is likely to interfere with the development of such skills [24,25]. Further, screen time through media use is likely to affect children’s behavior and capacity to pay attention through several mechanisms, as it may lead to sleep disturbances, which can adversely impact development. Media use at bedtime has been associated with increased autonomic activation due to hyperarousal, or disrupted melatonin production due to brightly lit screens [26,27]. Repeated exposure to violence and aggression through computer use (e.g., playing violent games or viewing violent media programs) can lead to aggressive and violent behavior [12,28]. Exposure to violent media also tends to increase anxiety and fear, as well as the acceptance of violence as an appropriate means for solving conflicts. Finally, children with higher levels of media use, including the computer and television, tend to be less physically active due to the sedentary nature of media use, increasing the risk of obesity [29,30,31].

Importantly, there are possibilities for bidirectional interactions between specific child characteristics and media use [32]. Children who may be considered more “difficult” are likely to be particularly vulnerable to increased exposure to media; for instance, children who have attentional problems may very well be attracted to technology because of the constant stimulation it provides [33,34,35,36].

As described above, although prior studies suggest that computer use, including home and school use, can impact on children’s development, there is limited evidence on the impact of portable and instantly accessible mobile devices, such as smartphones and tablets on child development. Mobile technology is relatively new and much of the gathered evidence is unclear or inconsistent. Mobile devices are replacing desktop computers, and their uses are highly diverse, including access to internet, games, applications, learning, online communication, and social networking sites. Therefore, in a rapidly changing era of digital technology, it is possible that using mobile devices like smartphones and tablets has a different impact on child adjustment compared with traditional media. In addition, early childhood is a pivotal period in various areas of development. Previous research has indicated that the preschool and early school years are a sensitive period for the acquisition of social competences and related abilities associated to social adjustment [37,38,39]. Therefore, the first year in elementary school (i.e., the transition period from preschool to elementary school) is an important developmental period during which children are expected to acquire prosocial abilities that will prepare them for social and emotional success. Therefore, the current study aimed to clarify the association between the use of mobile devices, such as smartphones and tablets, and emotional and behavioral problems in first-grade elementary school children.

## Materials and methods

### Participants

The present research was part of a longitudinal study examining the effects of the child-rearing environment on children’s social development and adjustment. Participants were all preschool children (*N* = 5,024) aged 5 years, recruited from in 52 kindergartens and 78 nursery schools in Nagoya city, a major urban area in Japan, in 2014. A total of 3,314 parents of preschool children provided written informed consent and agreed to participate at baseline in 2014. We plan to conduct a survey every year to follow up children from preschool to junior high school.

The current research took place in 2015, and self-report questionnaires were provided to the parents of 6-year-old children (*N* = 3,268) who were in first grade of elementary school (47 children had relocated). Children’s parents (*N* = 1,787) completed the questionnaires. Comparing the non-returning participants with the returning participants on demographic features, the non-returning participants tended to have relatively lower SES (i.e., family income, parental education level, and parental employment status) than did returning participants, meaning that there was a lower response rate of individuals with low SES compared to high SES (see S1 Table).

In the present study, in order to accurately clarify the association between mobile device use and child adjustment, children diagnosed with developmental problems and those whose parents did not return complete questionnaires were excluded from the analysis. For inclusion in the study, parents did not need to be the target child’s biological parents; however, they did need to reside with the child. Of the 1,787 children, 1,642 (91.9%) met the inclusion criteria.

### Ethics statement

Children’s parents were informed of the study purpose and procedures, and were made aware that they were not obligated to participate. The parents provided their written informed consent on behalf of their children prior to participating in this research. Ethical approval for this study was obtained from Kyoto University Ethics Committee (E2322).

### Measures

#### Outcome variable: Child adjustment

The Strengths and Difficulties Questionnaire (SDQ) is a 25-item measure of parents’ perceptions of their children’s prosocial and difficult behaviors [40]. The measure is categorized into five subscales: conduct problems (five items), hyperactivity/inattention (five items), emotional symptoms (five items), peer problems (five items), and prosocial behavior (five items). In the present study, the conduct problems, hyperactivity/inattention, emotional symptoms, and peer problems subscales were used to assess children’s emotional and behavioral problems. Items were rated on a 3-point Likert scale ranging from 0 (Not true) to 2 (Certainly true). The scale’s internal consistency and construct validity have been reported as adequate [41,42,43]. In the present study, Cronbach’s α coefficient for the SDQ ranged from .52 to .77 for the individual scales (conduct problems, 0.52; hyperactivity/inattention, 0.77; emotional symptoms, 0.68; peer problems, 0.61).

Considering the cut-off point for the Japanese version of the SDQ, we categorized participants into normal, borderline, and abnormal (or clinical) groups [43]. According to the cut-off score, the sample was categorized into an abnormal group when scoring above the 90th percentile (approximately 10%), a borderline group when scoring between the 80th and 90th percentile (approximately 10%), and a normal group when scoring below the 80th percentile (approximately 80%). However, to run the logistic regression with a bivariate outcome, we considered both the borderline and normal groups as the normal group.

#### Explanatory variable: Mobile device use

**Fig 1:**
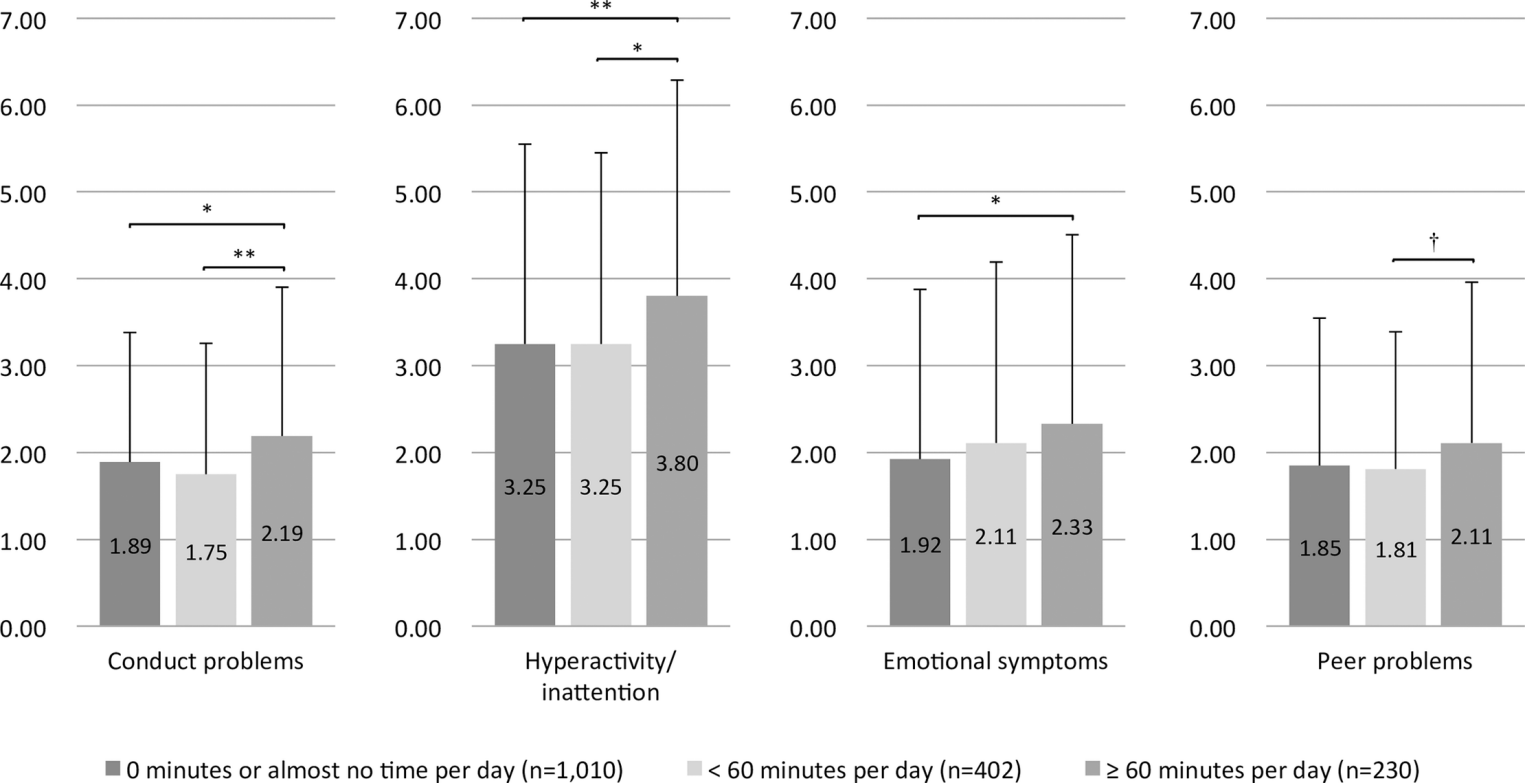
Mobile device use and emotional/behavioral problems (*N* = 1,642). † *p* < .10, * *p* < .05, ** *p* < .01, *** *p* < .001.

The explanatory variable in this study was children’s regular use of mobile devices, such as smartphones and tablets. Children’s use of mobile devices was assessed through average use time (in minutes) on a typical day. In this study, among 1,642 participants, 1,010 (61.5%) were non-users, 402 (24.5%) used devices less than 60 minutes on a typical day, and 230 (14.0%) used devices 60 minutes or more on a typical day. In terms of emotional/behavioral problems, users spending 60 minutes or more a day had significantly more problems/symptoms (i.e., conduct problems, hyperactivity/inattention, and emotional symptoms) compared to non-users or users spending less than 60 minutes a day (see Fig 1). Prior to selecting the cut-off point of 60 minutes, three different cut-off points (60 minutes, 90 minutes, and 120 minutes) had been considered. In order to identify the best cut-off points, we examined the sensitivity and specificity among the three possible cut-off points. The best cut-off point was 60 minutes, as it was characterized by highest combination of sensitivity and specificity among the three (see S2 Table). Therefore, in this study, when we ran a logistic regression with a bivariate explanatory variable, children using mobile devices less than an average of 60 minutes on a typical day were deemed to be “non-regular users,” and those with an average over 60 minutes on a typical day were considered “regular users”.

#### Covariates

Potential confounding variables were selected as covariates due to the differential chances of using mobile devices. Demographic variables included sex, presence of parents (two-parent family or single-parent family), and presence of siblings (presence or no presence of siblings). Socioeconomic status indicators included annual equalized household income (JPY) (less than 3 million JPY [approximately 30,000 USD], 3–6 million JPY [approximately 60,000 USD], 6–9 million JPY [approximately 90,000 USD], or 9 million JPY and more), maternal and paternal educational attainment (compulsory education [9 years], upper secondary school [10–12 years], up to 4 years at college/university [13–15 years], or more than 4 years at college/university [over 15 years]) and maternal and paternal employment status (employed [full-time], employed [part-time], or unemployed/homemaker). Parent/child interactions were measured through parent-report questionnaires that were collected during a 2015 survey. On this survey, parents were asked to report, in minutes, the average amount of time spent by both the mother and father talking or playing with children on a typical day. This variable was dichotomized into two group: parent/child interactions that lasted less than an average of 60 minutes a typical day and parent/child interactions that averaged over 60 minutes on a typical day. Past child temperament included children’s emotional/behavioral problems at preschool calculated using SDQ score at baseline in 2014 (normal/borderline group or abnormal group).

### Statistical analyses

First, mobile device use was evaluated according to children’s characteristics. Second, to address potential selection bias attributable to the differential chances of using mobile devices, a propensity score approach was used. The propensity score was calculated using variables supposed to potentially affect the use of mobile devices: sex, family composition (presence of parents and siblings), annual equalized household income, maternal and paternal educational attainment, maternal and paternal employment status, maternal and paternal average spending time of talking or playing with children, and children’s emotional/behavioral problems at preschool. Inverse probability of treatment weighted (IPTW) logistic regression analysis was then performed; the inverse of the propensity score was incorporated to the weighted logistic regression models to compute odds rate ratios (OR) for emotional/behavioral problems according to use mobile devices. This approach is an alternative to implementing propensity score matching to statistically balance confounding variables in non-randomized studies [44]. As several studies have suggested adverse impacts of non-educational media exposure on child development [45,46], we calculated the OR for emotional/behavioral problems based on whether or not children were using media for educational purposes, by performing logistic regressions as additional analyses. To estimate the effect of using media for educational purposes, we used explanatory variables categorized into “non-regular users,” “regular users including educational purposes,” and “regular users not including educational purposes.” These results are shown in Supporting Information (see S3 Table, S4 Table, S5 Table, and S6 Table). All statistical analyses were conducted using SPSS version 23.0.

## Results

### Study population

Results are shown regarding mobile device use and emotional/behavioral problems (Fig 1), and participant characteristics (Table 1). Users spending 60 minutes or more a day were categorized as regular users [230 (14.0%)], and non-users and users spending less than 60 minutes a day were categorized as non-regular users [1,412 (86.0%)]. On a typical day, regular users used mobile devices for approximately 1 hour and 20 minutes on average. Children’s average age was 6.88 years (*SD* = 0.35), and 51.2% were males (*n* = 841) and 48.8% females (*n* = 801). The mean ages of mothers and fathers were 38.29 (*SD* = 4.63) and 40.32 (*SD* = 5.46) years, respectively. The median annual household income was between 5 and 6 million JPY per year. On average, mothers and fathers had completed comparable years of education, 14.10 (*SD* = 1.77) and 14.55 (*SD* = 2.26) years, respectively. On average, mothers and fathers spent talking or playing with children for 230.41 (*SD* = 146.67) and 75.39 (*SD* = 77.54) minutes on typical day, respectively. The proportions of abnormal (or clinical) emotional/behavioral problems at preschool were 8.4% (*n* = 137).

**Table 1:** Participant characteristics (*N* = 1,642).

				Non-regular users (less than 60 minutes a day) *n* = 1,412		Regular users (60 minutes or more a day) *n* = 230		
		*n*	%	*n*	%	*n*	%	*p* -value
Sex								
	Female	801	48.8	712	50.4	89	38.7	.001
	Male	841	51.2	700	49.6	141	61.3	
Presence of parents								
	Two-parent family	1514	92.2	1302	92.2	212	92.2	.985
	Single-parent family	128	7.8	110	7.8	18	7.8	
Presence of siblings								
	Yes	1370	83.4	1180	83.6	190	82.6	.716
	No	272	16.6	232	16.4	40	17.4	
Annual household income (in millions of JPY)								
	≥ 9	276	17.3	242	17.6	34	15.2	.026
	6–9	458	28.6	406	29.5	52	23.2	
	3–6	704	44.0	599	43.5	105	46.9	
	< 3	162	10.1	129	9.4	33	14.7	
Maternal education level								
	More than 4 years at college/university	526	32.4	471	33.7	55	24.2	< .001
	Up to 4 years at college/university	674	41.5		586	41.9	88	38.8
	Upper secondary school	385	23.7	312	22.3	73	32.2	
	Compulsory education	40	2.5	29	2.1	11	4.8	
Paternal education level								
	More than 4 years at college/university	878	56.0	772	57.3	106	47.7	< .001
	Up to 4 years at college/university	233	14.9	200	14.8	33	14.9	
	Upper secondary school	381	24.3	323	24.0	58	26.1	
	Compulsory education	77	4.9	52	3.9	25	11.3	
Maternal employment status								
	Employed (full-time)	415	25.8	359	26.0	56	24.8	.485
	Employed (part-time)	542	33.7	458	33.1	84	37.2	
	Unemployed/homemaker	652	40.5	566	40.9	86	38.1	
Paternal employment status								
	Employed (full-time)	1527	98.0	1311	98.1	216	97.7	.821
	Employed (part-time)	27	1.7	23	1.7	4	1.8	
	Unemployed/homemaker	4	.3	3	.2	1	.5	
Maternal average spending time of talking or playing with children (minutes per day)								
	≥ 60	1520	94.5	1306	94.4	214	95.5	.474
	< 60	88	5.5	78	5.6	10	4.5	
Paternal average spending time of talking or playing with children (minutes per day)								
	≥ 60	858	58.1	731	57.6		127	60.5	.442
	< 60	620	41.9	537	42.4		83	39.5
Emotional/behavioral problems at preschool								
	Normal/borderline	1501	91.6	1301	92.3	200	87.3	.011
	Abnormal	137	8.4	108	7.7	29	12.7	

Strengths and Difficulties Questionnaire–Total Difficulties Score: normal/borderline: 0–15, abnormal: 16–40

A total of 61.3% of regular users were male, which was significantly higher than the proportion of males in the non-regular user group. Regarding annual household income, the proportion of lower-income families in the regular user group was significantly higher than in the non-regular user group. Regarding parental education level, the proportion of lower-education mothers and fathers in the regular user group was significantly higher than in the non-regular user group. Regarding children’s emotional/behavioral problems at preschool, the proportion classified as Abnormal in the regular user group was significantly higher than in the non-regular user group.

### Mobile device use among regular users

Regular users’ mobile device use was examined in relation to types of mobile devices (Table 2) and purpose of use (Table 3). Regarding mobile device types among regular users, 66.5% used their own mobile devices (smartphone: 9.1%, tablet: 16.1%, portable game device: 54.8%), and 94.3% used their parents’ mobile devices (smartphone: 74.3%, tablet: 46.5%, portable game device: 15.7%).

**Table 2:** Types of mobile devices (*N* = 230).

		*n*	%
Own mobile devices			
Children using own mobile devices		153	66.5
	Smartphone	21	9.1
	Tablet	37	16.1
	Portable game device (DS, PSP, etc.)	126	54.8
	Other	19	8.3
Parents’ mobile devices			
Children using parents’ mobile devices		217	94.3
	Smartphone	171	74.3
	Tablet	107	46.5
	Portable game device (DS, PSP, etc.)	36	15.7
	Other	72	33.2

**Table 3:** Purpose of mobile device use (*N* = 230).

	*n*	%
Viewing videos (YouTube, etc.)	179	77.8
Playing games	165	71.7
Taking and sharing pictures, figures, or photos	67	29.1
Learning/using applications related to education	42	18.3
Talking with friends, family, others	41	17.8
Using internet/searching for information	35	15.2
Sending and receiving messages (e-mail, Line, etc.)	24	10.4
Checking and informing of location	8	3.5
Other	3	1.3

Regarding mobile device use among regular users (Table 3), the main reported purposes were as follows; 77.8% reported viewing videos (YouTube, etc.); 71.7% playing games; 29.1% taking and sharing pictures, figures, or photos; 18.3% learning/using applications related to education; 17.8% talking with friends, family, others; 15.2% using internet/searching for information; and 10.4% sending and receiving messages (e-mail, Line, etc.).

### Association between mobile device use and child adjustment

The proportions of abnormal (or clinical) emotional/behavioral problems were as follows; conduct problems (elevated score: 5–10): non-regular users *n* = 79 (5.6%), regular users *n* = 24 (10.4%); hyperactivity/inattention (elevated score: 7–10): non-regular users *n* = 138 (9.8%), regular users *n* = 38 (16.5%); emotional symptoms (elevated score: 5–10): non-regular users *n* = 172 (12.2%), regular users *n* = 40 (17.4%); peer problems (elevated score: 5–10): non-regular users *n* = 115 (8.1%), regular users *n* = 26 (11.3%).

According to the logistic regression analysis, the crude OR for conduct problems relative to non-regular users was 1.99 (95% CI [1.23–3.22], *p* = .005) for regular users (Crude model in Table 4). The IPTW-OR for conduct problems was 1.77 (95% CI [1.03–3.04], *p* = .038) for regular users (IPTW model in Table 4).

**Table 4:** Association between mobile device use and conduct problems.

	Crude model				IPTW model			
	OR	95% CI		*p*-value	OR	95% CI		*p*-value
Non-regular users	Ref.				Ref.			
Regular users	1.99	1.23–3.22		.005	1.77	1.03–3.04		.038

Strengths and Difficulties Questionnaire–Conduct problems: normal/borderline: 0–4, abnormal: 5–10

IPTW = inverse probability of treatment weighted

The crude OR for hyperactivity/inattention relative to non-regular users was 1.85 (95% CI [1.25–2.74], *p* = .002) for regular users (Crude model in Table 5). The IPTW-OR for hyperactivity/inattention was 1.82 (95% CI [1.15–2.87], *p* = .009) for regular users (IPTW model in Table 5).

**Table 5:** Association between mobile device use and hyperactivity/inattention.

	Crude model				IPTW model			
	OR	95% CI		*p*-value	OR	95% CI		*p*-value
Non-regular users	Ref.				Ref.			
Regular users	1.85	1.25–2.74		.002	1.82	1.15–2.87		.009

Strengths and Difficulties Questionnaire–Hyperactivity/inattention: normal/borderline: 0–6, abnormal: 7–10

IPTW = inverse probability of treatment weighted

The crude OR for emotional symptoms relative to non-regular users was 1.54 (95% CI [1.06–2.24], *p* = .025) for regular users (Crude model in Table 6). The IPTW-OR for emotional symptoms was 1.53 (95% CI [0.99–2.43], *p* = .057) for regular users (IPTW model in Table 6).

**Table 6:** Association between mobile device use and emotional symptoms.

	Crude model				IPTW model			
	OR	95% CI		*p*-value	OR	95% CI		*p*-value
Non-regular users	Ref.				Ref.			
Regular users	1.54	1.06–2.24		.025	1.53	0.99–2.43		.057

Strengths and Difficulties Questionnaire–Emotional symptoms: normal/borderline: 0–4, abnormal: 5–10

IPTW = inverse probability of treatment weighted

Regular and non-regular users showed no significant differences in terms of peer problems (Crude model in Table 7). The IPTW-OR for peer problems was not significant for regular users (IPTW model in Table 7).

**Table 7:** Association between mobile device use and peer problems.

	Crude model				IPTW model			
	OR	95% CI		*p*-value	OR	95% CI		*p*-value
Non-regular users	Ref.				Ref.			
Regular users	1.46	0.93–2.28		.103	1.24	0.71–2.17		.452

Strengths and Difficulties Questionnaire–Peer problems: normal/borderline: 0–4, abnormal: 5–10

IPTW = inverse probability of treatment weighted

In addition, we calculated the OR for emotional/behavioral problems based on whether using media for educational purposes or not, by performing logistic regressions (see S3 Table, S4 Table, S5 Table, and S6 Table). Among regular users (*n* = 230), users using media with educational content were 18.3% (*n* = 42), and users using media that did not have educational content were 81.7% (*n* = 188). Relative to non-regular use, regular use of mobile devices without educational purpose was significantly linked to conduct problems (OR: 1.94, 95% CI [1.15–3.28], *p* = .014) and hyperactivity/inattention (OR: 1.85, 95% CI [1.20–2.85], *p* = .005), even after adjusting for covariates. Relative to non-regular use, regular use of mobile devices, including for educational purposes, was not significantly linked to any emotional/behavioral problems. Therefore, routine frequent use of mobile devices in the absence of educational content appears to be related to behavioral problems in childhood.

## Discussion

In the current study, we found that using mobile devices, such as smartphones and tablets, was associated with a higher likelihood of behavior problems (i.e., conduct problems and hyperactivity/inattention difficulties). Our analyses were conducted using the propensity score approach. We found that routine and frequent use of mobile devices without educational content is likely to be related to behavioral problems in childhood. Several mechanisms are likely to be involved in this relationship between mobile device use and the risk of emotional/behavioral problems.

First, frequent mobile device use is likely to increase children’s social isolation, and hinder opportunities for social interaction with family, friends, that benefits the development of social competence, resulting in emotional/behavioral problems. Previous research on children’s home computer use reported that more than half of the time children spend using computers is spent alone [47]. In addition, a study reported that children and adolescents spend 7–8 hours a day using a variety of media including television, video games, and computers, which is longer than they spend on any other activity [48]. Children can use mobile devices when and where they wish, and in turn, the use may become routinized and associated with personal space, which may further decrease children’s social interaction. Recently, children have had unprecedented access to new media. Although in some cases new media can foster communication and the generation of electronic relationships, it is also possible that the development and spread of new media devices may decrease children’s social interaction. Social interaction throughout childhood, primarily face-to-face, is a core factor impacting on the development of children’s social competence [49]. Especially, the development of social relationships with peers at home, school, and other contexts is a major achievement in childhood, and these interactions provide children with the foundation for social competence development [49,50,51]. Social competence in childhood gradually stabilizes over time, and is predictive of social adjustment and absence of psychopathology in later life [52,53,54,55]. Therefore, frequent use of mobile devices as well as computers might exacerbate children’s social deficits. However, research on the social effects of media technology use has produced mixed results including advantages and disadvantages. Some research on computer use indicates that moderate use does not significantly impact children’s social development or relationships with peers and family [56,57]. Furthermore, one study found that frequent computer game users interacted with peers outside school more often than did less frequent users [58]. In addition, internet use has been found to contribute to social well-being though the expansion of social networks [57]. Therefore, although the current study suggests that frequent mobile device use of more than 60 minutes on a typical day was linked to emotional and behavioral problems in first grade children, future studies should investigate in detail how much time is appropriate for children to spend using mobile devices.

There is a possibility that not only the quantity of time using mobile devices, but also the quality of use of mobile devices has influences on child development. In this study, we found that frequent mobile device use was significantly associated with higher externalizing problems (i.e., conduct problems and hyperactivity/inattention), but using mobile devices was not significantly associated with internalizing problems (i.e., emotional symptoms and peer problems). Although many applications and games for mobile devices do include content that encourages positive behaviors, such as cooperating and sharing, the content of numerous applications involves competition with aggression and violence. Much of the violence in media is often presented in either a sanitized and glamorized fashion, or with humor. A recent analysis of popular computer games found that more than half of all games contained aggression or violence [59,60]. Media which includes violent content is likely to be harmful for children’s development. Many studies have shown that repeated exposure to media violence, including television programs and films, increases children’s aggression and hostility [61,62]. In addition, several studies have suggested that playing a violent game can also lead to increased aggressiveness and hostility, and decreased social behavior [62]. Additionally, repeated exposure to media violence is likely to lead to anxiety and fear, aggressive thoughts, and the acceptance of violence as a primary means for solving conflict [63,64]. Thus, although the current study did not examine the content accessed by children with mobile devices, it is plausible that repeated exposure to violence in media and games though mobile devices might have an impact, which may be reflected in the association between frequent mobile device use and externalizing behavioral problems. Future studies should investigate in detail how specific content may impact on children using mobile devices.

Furthermore, in this study, we found that the proportion of frequent use of mobile devices was higher for children with lower SES families. This result is consistent with previous studies on other media use (e.g., television and videos) that lower SES children have the greatest amount of media exposure [65]. There is a possibility that factors other than using mobile devices may have influences on child development. Extensive literature has documented that socioeconomic disadvantage in childhood is related to both current and later impairment in mental health [66,67]. There are likely to be several pathways mediating the association between SES and child mental health. Many studies on the underlying psychological processes of how SES affects development have focused on parenting practices and parental investment. The first pathway is parenting practice. Lower SES families have more conflict and hostility, and the tendency of lower SES parents to engage in harsher and less responsive interactions with their children [68,69]. Studies on the family process model suggest that financial difficulties affect children’s socio-emotional development through the psychological well-being of parents and consequently their parenting strategies [70,71]. The second pathway is parental investment. Children with lower SES have less cognitively stimulating environments, such as fewer age-appropriate toys, fewer learning venues, and fewer educational materials [72]. Studies on the family investment model propose that families with higher SES are able to make significant investments in the development of their children, whereas more disadvantaged families must invest in more immediate family needs [73,74]. These investments involve several different dimensions of family support, including availability of learning materials, parental stimulation of learning both directly and through support of advanced or specialized training, the family’s standard of living, and residing in a location that fosters a child development. Furthermore, children with lower socioeconomic backgrounds are at a greater risk of higher chronic stress and higher risk of sleep problems, which negatively influences multiple aspects of health and well-being in children. Disadvantaged children must contend with a wide array of physical stressors and psychosocial stressors; as exposure to stressors accumulates, the chronic cumulative stressors strain and eventually damage their biological and psychological regulatory systems [75,76]. In addition, children in families with low SES have been found to have sleep problems, such as shorter and poorer-quality sleep [77,78]. Sleep problems are related to emotional and behavioral difficulties, possibly acting through hormonal, neuronal and psychological pathways [79,80]. Lower economic resources may make it more challenging for families to maintain children’s sleep environments that are quiet, dark, and kept at a comfortable temperature, and so children may experience greater difficulty falling asleep [81]. Children living in economically disadvantaged environments may have compromised sleep due to worries that prevents them from easily falling asleep. Economic disadvantage is associated with high levels of family stress and numerous specific stressors, including exposure to events that are unpredictable and uncontrollable, harsh discipline, and violence at home, school, or neighborhood [82]. Associations between cognitive arousal at bedtime, including worry, and sleep disturbance have been demonstrated in children. Sleep problems is a known predictor of emotional and behavioral problems and it is plausible that sleep problems may act as a mediator of the association between SES and poor health. Therefore, there is a possibility that factors regarding SES are likely to have influenced child development. Future research should incorporate data collection on such other potential factors.

In summary, the extent of the developmental effects of mobile device use is likely to depend on the amount of time spent and the content viewed by children. Frequent mobile device use is likely to increase children’s social isolation and hinder opportunities for social interaction, both of which promote social development. In addition, repeated exposure to violence in games and videos is likely to be harmful for child development. On the other hand, as mentioned earlier, media technology can also be beneficial to child development, for instance, by enhancing cognitive skills and academic performance. Therefore, parents are recommended to limit the amount of time that children spend using mobile devices, computers, and other media technology, and increase the opportunities for face-to-face interactions and playing with peers. In addition, parents are recommended to search for content that promotes building vocabulary, mathematical and science concepts, etc. We should recognize both the positive effects and potential harmful risks of mobile device use, including the advantages and disadvantages.

### Limitations

Our study has several limitations. First, an important issue affecting the interpretation of our data is the cross-sectional design. Although associations can be identified, causality cannot be inferred. Perhaps routine frequent use of mobile devices exposure causes behavioral problems, or perhaps children with behavioral problems are more attracted to routine frequent use of mobile devices. As mentioned earlier, there is the possibility of bidirectional associations between child social-emotional development and media use [32]. Indeed, more difficult children are likely to be particularly vulnerable to higher levels of media exposure [33,34,35,36]. Thus, longitudinal designs are needed to examine the effects of mobile device use on the later development and adjustment of children.

Second, there is a risk of selection bias. Although we used the IPTW approach, we could not consider unobservable factors influencing children’s use of mobile devices. For instance, the use of technology in different classrooms or schools might influence child technology-use behaviors. In addition, as mentioned earlier, in lower SES families, children are at a greater risk of exposure due to the lower quality of parenting style, lower investment, higher chronic stress, and higher sleep problems, etc. which negatively influence child development [70,71,72,73,74,75,76,77,78]. Future research should incorporate data on these other potential factors.

Third, we could not confirm the context of individual mobile device use. Repeated exposure to media violence is likely to increase children’s behavioral problems, such as aggression and hostility [61,62]. Thus, future studies should investigate not only the amount of time spent using mobile devices but also the context of use.

Finally, these findings may not be generalizable to all families, because there is a risk of attrition bias, and the sample was drawn from a limited geographical area in an urban metropolis of Japan. As mentioned earlier, the retention rate from the baseline survey to this survey was approximately 50%, and the returning participants tended to be relatively higher in SES than the non-returning participants. This indicates there is a risk of attrition bias. Therefore, there is the possibility that our analyses could not adequately evaluate the outcomes of mobile device use by children with lower SES, and our analyses may underestimate the influence of SES. The reproducibility of the current results should be confirmed using data from other regions in a variety of settings.

### Conclusions

Despite the above-mentioned limitations, our findings suggest that there is a risk that children’s routinized and frequent use of mobile devices is associated with emotional/behavioral problems. Excessive use of mobile devices, including smartphones and tablets, might interfere with children’s development in relation to social adjustment. Our findings suggest that preventing an excessive use of mobile devices may reduce the likelihood of behavioral problems in children. In this dynamic era of digital technology, both positive effects and potential harmful risks of mobile device use need to be recognized. Further research on the amount of time spent by children using these media and the viewed content is needed to help to maximize the positive effects and minimize the negative effects of mobile device use in children’s lives.

## Supporting information

S1 Table Parent and family characteristics of the study sample at baseline. (DOCX)

S2 Table Sensitivity and specificity rate of the model for three deferent cut-off points. (DOCX)

S3 Table Association between mobile device use and conduct problems. (DOCX)

S4 Table Association between mobile device use and hyperactivity/inattention. (DOCX)

S5 Table Association between mobile device use and emotional symptoms. (DOCX)

S6 Table Association between mobile device use and peer problems. (DOCX)

## References

[1] Strasburger VC, Hogan MJ. Policy statement: children, adolescents, and the media. Pediatrics. 2013;132: 958–961. 10.1542/peds.2013-2656 28448255

[2] Nielsen Company. Television, Internet and mobile usage in the U.S.: A2/M2 Three Screen Report. New York: Nielsen Company; 2009.

[3] Strasburger VC, Jordan AB, Donnerstein E. Health effects of media on children and adolescents. Pediatrics. 2010;125: 756–767. 10.1542/peds.2009-2563 20194281

[4] Vandewater EA, Rideout VJ, Wartella EA, Huang X, Lee JH, Shim MS. Digital childhood: electronic media and technology use among infants, toddlers, and preschoolers. Pediatrics. 2007;119: e1006–e1015. 10.1542/peds.2006-1804 17473074

[5] National Institute for Educational Policy Research. Zenkoku Gakuryoku Gakusyu Jyokyo Cyosa [Japanese]; 2014. Available from: http://www.nier.go.jp/14chousakekkahoukoku/

[6] Vandewater E, Lee S. Measuring children’s media use in the digital age: issues and challenges. Am Behav Sci. 2009;52: 1152–1176. 10.1177/0002764209331539 19763246 PMC2745155

[7] Li X, Atkins MS, Stanton B. Effects of home and school computer use on school readiness and cognitive development among head start children: a randomized controlled pilot trial. Merrill Palmer Quart. 2006;52: 239–263.

[8] Fiorini M. The effect of home computer use on children's cognitive and non-cognitive skills. Econ Educ Rev. 2010;29: 55–72. 10.1016/j.econedurev.2009.06.006

[9] Rocheleau B. Computer use by school-age children: trends, patterns and predictors. J Educ Comput Res. 1995;12: 1–17. 10.2190/MHUR-4FC9-B187-T8H4

[10] Nichols LM. The influence of student computer-ownership and in-home use on achievement in an elementary school computer programming curriculum. J Educ Comput Res. 1992;8: 407–421. 10.2190/6UNE-05L9-KCRH-D1NQ

[11] Anderson D, Subramanyam R. The new digital American family: Understanding family dynamics, media, and purchasing behavior trends New York: The Nielson Company; 2011.

[12] Subrahmanyam K, Kraut RE, Greenfield PM, Gross EF. The impact of home computer use on children’s activities and development. Future Child. 2000;10: 123–144. 11255703

[13] Griffiths MD. Friendship and social development in children and adolescents: the impact of electronic technology. Educ Child Psychol. 1997;14: 25–37.

[14] Amichai-Hamburger Y, Ben-Artzi E. Loneliness and Internet use. Comput Human Behav. 2003;19: 71–80. 10.1016/S0747-5632(02)00014-6

[15] Rikkers W, Lawrence D, Hafekost J, Zubrick SR. Internet use and electronic gaming by children and adolescents with emotional and behavioural problems in Australia—results from the second child and adolescent survey of mental health and wellbeing. BMC Public Health. 2016;16: 399 10.1186/s12889-016-3058-1 27178325 PMC4866411

[16] Huston AC, Wright JC, Marquis J, Green SB. How young children spend their time: Television and other activities. Dev Psychol. 1999;35: 912–925. 10.1037/0012-1649.35.4.912 10442861

[17] Tomopoulos S, Valdez PT, Dreyer BP, Fierman AH, Berkule SB, Kuhn M, et al Is exposure to media intended for preschool children associated with less parent–child shared reading aloud and teaching activities? Ambul Pediatr. 2007;7: 18–24. 10.1016/j.ambp.2006.10.005 17261478

[18] Plowman L, McPake J, Stephen C. The technologisation of childhood? Young children and technology in the home. Children & Society. 2010;24: 63–74. 10.1111/j.1099-0860.2008.00180.x

[19] Shimpi PM, Huttenlocher J. Redirective labels and early vocabulary development. J Child Lang. 2007;34: 845–859. 18062361 10.1017/s0305000907008112

[20] Landry SH, Miller-Loncar CL, Smith KE, Swank PR. The role of early parenting in children’s developmental and executive processes. Dev Neuropsychol. 2002:21: 15–41. 10.1207/S15326942DN2101_2 12058834

[21] NICHD Early Child Care Research Network. Predicting individual differences in attention, memory, and planning in first graders from experience at home, child care and school. Dev Psychol. 2005;41: 99–114. 10.1037/0012-1649.41.1.99 15656741

[22] Hart B. A natural history of early language experience. Topics Early Child Spec Educ. 2000;20: 28–32. 10.1177/027112140002000105

[23] Ginsburg KR; American Academy of Pediatrics Committee on Communications; American Academy of Pediatrics Committee on Psychosocial Aspects of Child and Family Health. The importance of play in promoting healthy child development and maintaining strong parent-child bonds. Pediatrics. 2007;119: 182–191. 10.1542/peds.2006-2697 17200287

[24] Vandewater EA, Bickham DS, Lee JH. Time well spent? Relating television use to children’s free-time activities. Pediatrics. 2006;117: e181–e191. 10.1542/peds.2005-0812 16452327 PMC2862999

[25] Schmidt ME, Pempek TA, Kirkorian HL, Lund AF, Anderson DR. The effects of background television on the toy play behavior of very young children. Child Dev. 2008;79: 1137–1151. 10.1111/j.1467-8624.2008.01180.x 18717911

[26] Kubota T, Uchiyama M, Suzuki H, Shibui K, Kim K, Tan X, et al Effects of nocturnal bright light on saliva melatonin, core body temperature and sleep propensity rhythms in human subjects. Neurosci Res. 2002;42: 115–122. 10.1016/S0168-0102(01)00310-8 11849730

[27] Higuchi S, Motohashi Y, Liu Y, Maeda A. Effects of playing a computer game using a bright display on presleep physiological variables, sleep latency, slow wave sleep and REM sleep. J Sleep Res. 2005;14: 267–273. 10.1111/j.1365-2869.2005.00463.x 16120101

[28] Ferguson CJ, Olson CK. Video game violence use among "vulnerable" populations: The impact of violent games on delinquency and bullying among children with clinically elevated depression or attention deficit symptoms. J Youth Adolesc. 2014;43: 127–136. 10.1007/s10964-013-9986-5 23975351

[29] Dennison BA, Erb TA, Jenkins PL. Television viewing and television in bedroom associated with overweight risk among low-income preschool children. Pediatrics. 2002;109: 1028–1035. 10.1542/peds.109.6.1028 12042539

[30] Bremer J. The internet and children: advantages and disadvantages. Child Adolesc Psychiatr Clin N Am. 2005;14: 405–428. 10.1016/j.chc.2005.02.003 15936666

[31] de Jong E, Visscher TLS, HiraSing RA, Heijmans MW, Seidell JC, Renders CM. Association between TV viewing, computer use and overweight, determinants and competing activities of screen time in 4- to 13-year-old children. Int J Obes (Lond). 2013;37: 47–53. 10.1038/ijo.2011.244 22158265

[32] Valkenburg PM, Peter J. The differential susceptibility to media effects model. J Commun. 2013;63: 221–243. 10.1111/jcom.12024

[33] Radesky JS, Peacock-Chambers E, Zuckerman B, Silverstein M. Use of mobile technology to calm upset children: Associations with social-emotional development. JAMA Pediatr. 2016;170: 397–399. 10.1001/jamapediatrics.2015.4260 26928293

[34] Thompson AL, Adair LS, Bentley ME. Maternal characteristics and perception of temperament associated with infant TV exposure. Pediatrics. 2013;131: e390–e397. 10.1542/peds.2012-1224 23296440 PMC3557404

[35] Schmidt ME, Rich M, Rifas-Shiman SL, Oken E, Taveras EM. Television viewing in infancy and child cognition at 3 years of age in a US cohort. Pediatrics. 2009;123: e370–e375. 10.1542/peds.2008-3221 19254972 PMC4042392

[36] Huesmann LR, Eron LD, Klein R, Brice P, Fischer P. Mitigating the imitation of aggressive behaviors by changing children’s attitudes about media violence. J Pers Soc Psychol. 1983;44: 899–910. 6864445 10.1037//0022-3514.44.5.899

[37] Watson AC, Nixon CL, Wilson A, Capage L. Social interaction skills and theory of mind in young children. Dev Psychol. 1999;35: 386–391. 10.1037/0012-1649.35.2.386 10082009

[38] Cole PM, Teti LO, Zahn-Waxler C. Mutual emotion regulation and the stability of conduct problems between preschool and early school age. Dev Psychopathol. 2003;15: 1–18. 10.1017/S0954579403000014 12848432

[39] Stright AD, Gallagher KC, Kelley K. Infant temperament moderates relations between maternal parenting in early childhood and children’s adjustment in first grade. Child Dev. 2008;79: 186–200. 10.1111/j.1467-8624.2007.01119.x 18269517

[40] Goodman R. The strengths and difficulties questionnaire: a research note. J Child Psychol Psychiatry. 1997;38: 581–586. 10.1111/j.1469-7610.1997.tb01545.x 9255702

[41] Goodman R. The extended version of the Strengths and Difficulties Questionnaire as a guide to child psychiatric caseness and consequent burden. J Child Psychol Psychiatry. 1999;40: 791–799. 10.1111/1469-7610.00494 10433412

[42] Goodman R, Ford T, Simmons H, Gatward R, Meltzer H. Using the Strengths and Difficulties Questionnaire (SDQ) to screen for child psychiatric disorders in a community sample. Br J Psychiatry. 2000;177: 534–539. 11102329 10.1192/bjp.177.6.534

[43] Matsuishi T, Nagano M, Araki Y, Tanaka Y, Iwasaki M, Yamashita Y, et al Scale properties of the Japanese version of the Strengths and Difficulties Questionnaire (SDQ): a study of infant and school children in community samples. Brain Dev. 2008;30: 410–415. 10.1016/j.braindev.2007.12.003 18226867

[44] Robins JM, Hernán MA, Brumback B. Marginal structural models and causal inference in epidemiology. Epidemiology. 2000;11: 550–560. 10955408 10.1097/00001648-200009000-00011

[45] Manganello JA, Taylor CA. Television exposure as a risk factor for aggressive behavior among 3-year-old children. Arch Pediatr Adolesc Med. 2009;163: 1037–1045. 10.1001/archpediatrics.2009.193 19884595

[46] Zimmerman FJ, Christakis DA. Children’s television viewing and cognitive outcomes: A longitudinal analysis of national data. Arch Pediatr Adolesc Med. 2005;159: 619–625. 10.1001/archpedi.159.7.619 15996993

[47] Roberts DF. Kids and media at the new millennium Pennsylvania: DIANE Publishing; 1999.

[48] Rideout V, Foehr UG, Roberts DF. Generation M2: Media in the lives of 8- to 18-year-olds Menlo Park, CA: Kaiser Family Foundation; 2010.

[49] Dworetzky JP. Introduction to Child development. 6th ed. California: Wadsworth Publishing; 1996

[50] Guralnick MJ. Family and child influences on the peer-related social competence of young children with developmental delays. Dev Dis Res Rev. 1999;5: 21–29. 10.1002/(SICI)1098-2779(1999)5:1<21::AID-MRDD3>3.0.CO;2-O

[51] Coie JD, Dodge KA. Multiple sources of data on social behavior and social status in the school: a cross-age comparison. Child Dev. 1988;59: 815–829. 10.2307/1130578 3383681

[52] Eisenberg N, Fabes R, Spinrad TL. Prosocial development: Social, emotional, and personality development In: Damon W, Eisenberg N, editors. Handbook of child psychology: vol. 3 5th ed. New York: John Wiley; 1998 pp. 701–778.

[53] McClelland MM, Morrison FJ. The emergence of learning-related social skills in preschool children. Early Child Res Q. 2003;18: 206–224. 10.1016/S0885-2006(03)00026-7

[54] Campbell S. Hard-to-manage preschool boys: externalizing behavior, social competence, and family context at two-year followup. J Abnorm Child Psychol. 1994;22: 147–166. 10.1007/BF02167897 8064027

[55] Coie JD, Dodge KA. Multiple sources of data on social behavior and social status in the school: a cross-age comparison. Child Dev. 1988;59: 815–829. 10.2307/1130578 3383681

[56] Phillips CA, Rolls S, Rouse A, Griffiths MD. Home video game playing in schoolchildren: a study of incidence and patterns of play. J Adolesc. 1995;18: 687–691. 10.1006/jado.1995.1049

[57] Jackson LA. Adolescents and the Internet In: Romer D, Jamieson P, editors. The changing portrayal of American youth in popular media. Annenberg Public Policy Center at the University of Pennsylvania. New York: Oxford University Press; 2008 pp. 377–410.

[58] Colwell J, Grady C, Rhaiti S. Computer games, self esteem, and gratification of needs in adolescents. J Community Appl Soc Psychol. 1995;5: 195–206. 10.1002/casp.2450050308

[59] Anderson CA, Gentile DA, Buckley KE. Violent video game effects on children and adolescents: Theory, research, and public policy New York: Oxford University Press; 2007.

[60] Dietz TL. An examination of violence and gender role portrayals in video games: implications for gender socialization and aggressive behavior. Sex Roles. 1998;38: 425–442. 10.1023/A:1018709905920

[61] Zillmann D, Weaver JB III. Psychoticism in the effect of prolonged exposure to gratuitous media violence on the acceptance of violence as a preferred means of conflict resolution. Pers Individ Dif. 1997;22: 613–627. 10.1016/S0191-8869(96)00245-0

[62] Ybarra ML, Diener-West M, Markow D, Leaf PJ, Hamburger M, Boxer P. Linkages between Internet and other media violence with seriously violent behavior by youth. Pediatrics. 2008;122: 929–937. 10.1542/peds.2007-3377 18977970

[63] Anderson CA, Berkowitz L, Donnerstein E, Huesmann LR, Johnson JD, Linz D, et al The influence of media violence on youth. Psychol Sci Public Interest. 2002;4: 81–110. 10.1111/j.1529-1006.2003.pspi_1433.x 26151870

[64] Bushman BJ, Anderson CA. Comfortably numb: desensitizing effects of violent media on helping others. Psychol Sci. 2009;20: 273–277. 10.1111/j.1467-9280.2009.02287.x 19207695

[65] Certain LK, Kahn RS. Prevalence, correlates, and trajectory of television viewing among infants and toddlers. Pediatrics. 2002;109: 634–642. 10.1542/peds.109.4.634 11927708

[66] Bradley RH, Corwyn RF. Socioeconomic status and child development. Annu Rev Psychol. 2002;53: 371–399. 10.1146/annurev.psych.53.100901.135233 11752490

[67] Reiss F. Socioeconomic inequalities and mental health problems in children and adolescents: a systematic review. Soc Sci Med. 2013;90, 24–31. 10.1016/j.socscimed.2013.04.026 23746605

[68] Conger RD, Donnellan MB. An interactionist perspective on the socioeconomic context of human development. Annu Rev Psychol. 2007;58: 175–199. 10.1146/annurev.psych.58.110405.085551 16903807

[69] Grant KE, Compas BE, Stuhlmacher AF, Thurm AE, McMahon SD, Halpert JA. Stressors and child and adolescent psychopathology: Moving from markers to mechanisms of risk. Psychol Bull. 2003;129: 447–466. 10.1037/0033-2909.129.3.447 12784938

[70] Conger RD, Conger KJ. Resilience in Midwestern families: selected findings from the first decade of a prospective, longitudinal study. J Marriage Fam. 2002;64: 361–373. 10.1111/j.1741-3737.2002.00361.x

[71] Hosokawa R, Katsura T. A longitudinal study of socioeconomic status, family processes, and child adjustment from preschool until early elementary school: the role of social competence. Child Adolesc Psychiatr Ment Health. 2017;11: 62 10.1186/s13034-017-0206-z 29270216 PMC5738164

[72] Evans GW. The environment of childhood poverty. Am Psychol. 2004;59: 77–92. 10.1037/0003-066X.59.2.77 14992634

[73] Linver MR, Brooks-Gunn J, Kohen D. Family processes as pathways from income to young children’s development. Dev. Psychol. 2002;38: 719–734. 10.1037/0012-1649.38.5.719 12220050

[74] Mayer SE. What Money Can’t Buy: Family Income and Children’s Life Chances. Massachusetts: Harvard University Press; 1997.

[75] Evans GW, Kim P. Childhood poverty, chronic stress, self‐regulation, and coping. Child Dev Perspect. 2013;7: 43–48. 10.1111/cdep.12013

[76] Kim P, Evans GW, Angstadt M, Ho SS, Sripada CS, Swain JE, et al Effects of childhood poverty and chronic stress on emotion regulatory brain function in adulthood. Proc Natl Acad Sci USA 2013;110: 18442–18447. 10.1073/pnas.1308240110 24145409 PMC3831978

[77] Bøe T, Hysing M, Stormark KM, Lundervold AJ, Sivertsen B. Sleep problems as a mediator of the association between parental education levels, perceived family economy and poor mental health in children. J Psychosom Res 2012;73: 430–436. 10.1016/j.jpsychores.2012.09.008 23148810

[78] El-Sheikh M, Bagley EJ, Keiley M, Elmore-Staton L, Chen E, Buckhalt JA. Economic adversity and children’s sleep problems: multiple indicators and moderation of effects. Health Psychol. 2013;32: 849–859. 10.1037/a0030413 23148451 PMC4008970

[79] Gregory AM, Sadeh A. Sleep emotional and behavioral difficulties in children and adolescents. Sleep Med Rev. 2012;16: 129–136. 10.1016/j.smrv.2011.03.007 21676633

[80] Van Cauter E, Spiegel K. Sleep as a mediator of the relationship between socioeconomic status and health: a hypothesis. Ann N Y Acad Sci. 1999;896: 254–261. 10.1111/j.1749-6632.1999.tb08120.x 10681902

[81] Milan S, Snow S, Belay S. The context of preschool children’s sleep: racial/ethnic differences in sleep locations, routines, and concerns. J Fam Psychol. 2007;21: 20–28. 10.1037/0893-3200.21.1.20 17371106

[82] Miller GE, Chen E. The biological residue of childhood poverty. Child Dev Perspect. 2013;7: 67–73. 10.1111/cdep.12021 24032051 PMC3766848

