# Correction: Association between mobile technology use and child adjustment in early elementary school age

**DOI:** 10.1371/journal.pone.0208844

**Published:** 2018-12-14

**Authors:** The PLOS ONE Staff

In Table 1, some values appear in the incorrect columns. Please see the corrected Table 1 here.

**Table 1:** Participant characteristics (N = 1,642).

			Non-regular users (less than 60 minutes a day) *n* = 1,412		Regular users (60 minutes or more a day) *n* = 230		
	*n*	%	*n*	%	*n*	%	*p* -value
Sex							
Female	801	48.8	712	50.4	89	38.7	.001
Male	841	51.2	700	49.6	141	61.3	
Presence of parents							
Two-parent family	1514	92.2	1302	92.2	212	92.2	.985
Single-parent family	128	7.8	110	7.8	18	7.8	
Presence of siblings							
Yes	1370	83.4	1180	83.6	190	82.6	.716
No	272	16.6	232	16.4	40	17.4	
Annual household income (in millions of JPY)							
≥ 9	276	17.3	242	17.6	34	15.2	.026
6–9	458	28.6	406	29.5	52	23.2	
3–6	704	44.0	599	43.5	105	46.9	
< 3	162	10.1	129	9.4	33	14.7	
Maternal education level							
More than 4 years at college/university	526	32.4	471	33.7	55	24.2	< .001
Up to 4 years at college/university	674	41.5	586	41.9	88	38.8	
Upper secondary school	385	23.7	312	22.3	73	32.2	
Compulsory education	40	2.5	29	2.1	11	4.8	
Paternal education level							
More than 4 years at college/university	878	56.0	772	57.3	106	47.7	< .001
Up to 4 years at college/university	233	14.9	200	14.8	33	14.9	
Upper secondary school	381	24.3	323	24.0	58	26.1	
Compulsory education	77	4.9	52	3.9	25	11.3	
Maternal employment status							
Employed (full-time)	415	25.8	359	26.0	56	24.8	.485
Employed (part-time)	542	33.7	458	33.1	84	37.2	
Unemployed/homemaker	652	40.5	566	40.9	86	38.1	
Paternal employment status							
Employed (full-time)	1527	98.0	1311	98.1	216	97.7	.821
Employed (part-time)	27	1.7	23	1.7	4	1.8	
Unemployed/homemaker	4	.3	3	.2	1	.5	
Maternal average spending time of talking or playing with children (minutes per day)							
≥ 60	1520	94.5	1306	94.4	214	95.5	.474
< 60	88	5.5	78	5.6	10	4.5	
Paternal average spending time of talking or playing with children (minutes per day)							
≥ 60	858	58.1	731	57.6	127	60.5	.442
< 60	620	41.9	537	42.4	83	39.5	
Emotional/behavioral problems at preschool							
Normal/borderline	1501	91.6	1301	92.3	200	87.3	.011
Abnormal	137	8.4	108	7.7	29	12.7	

Strengths and Difficulties Questionnaire–Total Difficulties Score: normal/borderline: 0–15, abnormal: 16–40
