# Supplementary material for: Association between mobile technology use and child adjustment in early elementary school age
Source: PLoS One. 2018 Jul 25;13(7):e0199959. doi: 10.1371/journal.pone.0199959 (PMC6059409; doi:10.1371/journal.pone.0199959)
Supplement: S1 Table — (DOCX) [file pone.0199959.s001.docx]

| **S1 Table. Parent and Family Characteristics of the Study Sample at Baseline.** | | | | | | | |
| --- | --- | --- | --- | --- | --- | --- | --- |
|  |  | Returning participants (Follow-up group) | |  | Non-returning participants  (Drop-out group) | |  |
|  |  | *n* | % |  | *n* | % | *p*-value |
| Annual household income (in millions of JPY) | |  |  |  |  |  |  |
|  | ≥ 9 | 286 | 17.1 |  | 190 | 12.6 | < .001 |
|  | 6–9 | 476 | 28.5 |  | 356 | 23.6 |  |
|  | 3–6 | 734 | 44.0 |  | 708 | 46.9 |  |
|  | < 3 | 172 | 10.3 |  | 257 | 17.0 |  |
| Maternal education level | |  |  |  |  |  |  |
|  | More than 4 years at college/university | 549 | 32.4 |  | 373 | 24.2 | < .001 |
|  | Up to 4 years at college/university | 696 | 41.1 |  | 608 | 39.4 |  |
|  | Upper secondary school | 409 | 24.1 |  | 470 | 30.5 |  |
|  | Compulsory education | 41 | 2.4 |  | 92 | 6.0 |  |
| Paternal education level | |  |  |  |  |  |  |
|  | More than 4 years at college/university | 913 | 55.8 |  | 631 | 44.5 | < .001 |
|  | Up to 4 years at college/university | 243 | 14.9 |  | 240 | 16.9 |  |
|  | Upper secondary school | 398 | 24.3 |  | 426 | 30.0 |  |
|  | Compulsory education | 81 | 5.0 |  | 121 | 8.5 |  |
| Maternal employment status | |  |  |  |  |  |  |
|  | Employed (full-time) | 438 | 26.1 |  | 368 | 24.1 | < .001 |
|  | Employed (part-time) | 560 | 33.4 |  | 653 | 42.7 |  |
|  | Unemployed/homemaker | 681 | 40.6 |  | 508 | 33.2 |  |
| Paternal employment status | |  |  |  |  |  |  |
|  | Employed (full-time) | 1589 | 98.0 |  | 1383 | 97.9 | .139 |
|  | Employed (part-time) | 28 | 1.7 |  | 20 | 1.4 |  |
|  | Unemployed/homemaker | 4 | .2 |  | 10 | .7 |  |
