# Supplementary material for: Association between mobile technology use and child adjustment in early elementary school age
Source: PLoS One. 2018 Jul 25;13(7):e0199959. doi: 10.1371/journal.pone.0199959 (PMC6059409; doi:10.1371/journal.pone.0199959)
Supplement: S2 Table — (DOCX) [file pone.0199959.s002.docx]

| **S2 Table. Sensitivity and Specificity Rate of the Model for Three Deferent Cut-off Points.** | | | |
| --- | --- | --- | --- |
|  | | Sensitivity | Specificity |
| Cut-off point | | % | % |
| Conduct problems | |  |  |
|  | 60 minute | 23.3 | 86.6 |
|  | 90 minute | 4.9 | 96.0 |
|  | 120 minute | 1.9 | 96.7 |
| Hyperactivity/Inattention | |  |  |
|  | 60 minute | 21.6 | 86.9 |
|  | 90 minute | 5.7 | 96.2 |
|  | 120 minute | 4.5 | 96.9 |
| Emotional Symptoms | |  |  |
|  | 60 minute | 18.9 | 86.7 |
|  | 90 minute | 4.7 | 96.1 |
|  | 120 minute | 1.9 | 96.6 |
| Peer Problems | |  |  |
|  | 60 minute | 18.4 | 86.4 |
|  | 90 minute | 7.8 | 96.3 |
|  | 120 minute | 5.7 | 97.0 |
