# Supplementary material for: Association between mobile technology use and child adjustment in early elementary school age
Source: PLoS One. 2018 Jul 25;13(7):e0199959. doi: 10.1371/journal.pone.0199959 (PMC6059409; doi:10.1371/journal.pone.0199959)
Supplement: S6 Table — (DOCX) [file pone.0199959.s006.docx]

| **S6 Table. Association between Mobile Device Use and Peer Problems.** | | | | | | |
| --- | --- | --- | --- | --- | --- | --- |
|  | Model 1 | | | Model 2 | | |
|  | Crude OR | 95% CI | *p*-value | Adjusted OR ^a^ | 95% CI | *p*-value |
| Non-regular users | Ref. |  |  | Ref. |  |  |
| Regular users including educational purposes | 1.29 | 0.77–2.15 | .332 | 1.10 | 0.65–1.88 | .725 |
| Regular users not including educational purposes | 2.25 | 0.97–5.18 | .057 | 2.19 | 0.92–5.17 | .074 |
| Strengths and Difficulties Questionnaire–Peer problems: normal/borderline: 0–4, abnormal: 5–10  ^a^ Odds ratio adjusted for sex, family composition (presence of parents and siblings), annual equalized household income, maternal and paternal educational attainment, maternal and paternal employment status, maternal and paternal average spending time of talking or playing with children, and children’s emotional/behavioral problems at preschool. | | | | | | |
